# Stability of conditioned pain modulation in two musculoskeletal pain models: investigating the influence of shoulder pain intensity and gender

**DOI:** 10.1186/1471-2474-14-182

**Published:** 2013-06-10

**Authors:** Carolina Valencia, Lindsay L Kindler, Roger B Fillingim, Steven Z George

**Affiliations:** 1 Department of Applied Medicine and Rehabilitation, Indiana State University, Terre Haute, IN, USA; 2 School of Nursing, University of Portland, Portland, OR, USA; 3 Department of Community Dentistry and Behavioral Science, University of Florida, Gainesville, FL, USA; 4 Department of Physical Therapy, University of Florida, UFHSC, Box 100154, Gainesville, FL 32610 - 0154, USA; 5 Center for Pain Research and Behavioral Health, University of Florida, Gainesville, FL, USA

**Keywords:** Conditioned pain modulation, Stability, Shoulder pain

## Abstract

**Background:**

Several chronic pain populations have demonstrated decreased conditioned pain modulation (CPM). However there is still a need to investigate the stability of CPM paradigms before the measure can be recommended for implementation. The purpose of the present study was to assess whether shoulder pain intensity and gender influence CPM stability within and between sessions.

**Methods:**

This study examined two different musculoskeletal pain models, clinical shoulder pain and an experimental model of shoulder pain induced with eccentric exercise in healthy participants. Patients in the clinical cohort (N = 134) were tested before surgery and reassessed 3 months post-surgery. The healthy cohort (N = 190) was examined before inducing pain at the shoulder, and 48 and 96 hours later.

**Results:**

Our results provide evidence that 1) stability of inhibition is not related to changes in pain intensity, and 2) there are sex differences for CPM stability within and between days.

**Conclusions:**

Fluctuation of pain intensity did not significantly influence CPM stability. Overall, the more stable situations for CPM were females from the clinical cohort and males from the healthy cohort.

## Background

Various quantitative sensory testing (QST) modalities have been used to assess particular mechanisms of pain perception in healthy individuals and those with chronic pain. Conditioned pain modulation (CPM) is one QST methodology with purported clinical relevance [1,2] and potential to predict the development of chronic pain [3]. This test paradigm has been previously described as diffuse noxious inhibitory controls but a recent consensus [4] concluded that the term CPM more accurately describes the testing phenomenon being observed. In short, CPM uses a “pain inhibits pain” test paradigm to activate the descending endogenous analgesia system [5].

It is believed that one noxious conditioning stimulus inhibits pain from another noxious test stimulus by activating a spinal-supraspinal-spinal loop, resulting in functional inhibitory pain modulation [1]. The inability of the noxious conditioning stimulus to decrease the pain intensity of the noxious testing stimulus indicates a potential deficiency in the body’s endogenous pain modulatory ability. Several chronic pain populations, including fibromyalgia [6], tempromandibular joint disorder [7], and irritable bowel syndrome [8], have demonstrated decreased CPM efficiency. These results, along with others showing the association of CPM with the development of chronic pain [3], resolution of pain following treatment [9], and ability to predict clinical pain [1], has led researchers to advocate for CPM as a clinically relevant QST modality [10].

Recently, efforts have gone into standardizing procedures used to assess CPM [4,5,11]; however there is still a need to investigate factors that influence CPM stability. One investigation that examined the test retest reliability of CPM in a small sample of healthy subjects found high reliability among repeated testing within a single session [11], however the exploration of CPM reliability in different musculoskeletal pain models has not yet been described. This is an important issue which would have implications for use of this measure in clinical populations. Since the evidence shows a dysfunction of endogenous analgesia system among chronic pain groups [12], it is reasonable to think that CPM reliability could be affected by having the system already engaged with the presence of shoulder pain. Furthermore, since sex differences in pain related responses to experimental pain measures have also been reported [13,14], the effect of sex on reliability of CPM also has clinical implications.

Therefore, the present study used two different musculoskeletal pain models to assess whether presence of shoulder pain and gender influence CPM stability within and between sessions. To serve this purpose, this study examined patients with acute and sub-acute shoulder pain preparing to undergo shoulder surgery, and healthy subjects with exercise induced muscle pain (EIMP) at the shoulder [15,16]. Understanding how shoulder pain and gender affect pain inhibitory responses, and estimating the error associated with this commonly used measure, will add to our understanding of CPM providing clinical relevance for this particular experimental pain measure.

## Methods

### Subjects

The University of Florida’s institutional review board for human participants approved this study. This prospective design was part of a larger study and includes two groups of participants, a clinical cohort of patients having shoulder surgery, and a healthy cohort. All participants provided informed consent before participating in this study.

#### Clinical cohort (clinical pain model)

This study includes data from consecutive patients seeking treatment of shoulder disorder, which were recruited from University of Florida’s Orthopedics Sports Medicine Institute (OSMI). The inclusion criteria for being a participant in the clinical cohort were: patient between 18 and 85 years of age, complaints of pain limited to anterior, lateral, or posterior shoulder, rotator cuff tendinopathy, adhesive capsulitis, SLAP (Superior Labrum from Anterior to Posterior) lesion, and scheduled for arthroscopic surgery. Exclusion criteria for the prospective clinical cohort were: current complaints of pain greater than the past 3 months involving neck, elbow, hand, low back, hip, knee, or ankle, massive or complete rotator cuff tear, shoulder OA or RA, prior shoulder surgery within the past year, current shoulder fracture, tumor, or infection, previously diagnosed chronic pain disorder, current psychiatric management, and gastrointestinal or renal illness [17].

#### Healthy cohort (acute pain model)

Healthy volunteers were recruited via advertisements from the University and local community. The inclusion criteria for the healthy cohort were: healthy subjects (without any pain or psychological condition) between 18 and 85 years of age, and English speaking. The exclusion criteria were: history of neck or shoulder injury, sensory or motor impairment of the shoulder, regular or recent participation in high or low intensity upper-extremity weight training, or currently taking pain medication. These eligibility criteria have been used in our previous studies of exercise-induced shoulder pain [15,16].

### Measures and procedure

#### Demographic information

Study participants completed a standard intake information form. Demographic data collected at initial evaluation included gender, age, race, ethnicity, employment status, marital status, and educational level.

#### Conditioned pain modulation (CPM)

**Test stimulus** Suprathreshold heat pain response (SHPR) was used as the test stimulus. Pathway Pain & Sensory Evaluation System (Medoc, Ramat Yishai, Israel), with a thermode of 2.5 cm^2^ surface area was used. Sequences of 5 consecutive heat pulses of < 1-second duration at interpulse intervals of 0.33 Hz were delivered to the thenar eminence of the non-surgical side for the clinical cohort, and non-dominant side for the healthy cohort, as previously described [18-20]. The temperature used for the test stimulus was determined from a previous SHPR assessment (five minutes before CPM assessment). One series of five stimuli was applied at each of three different target temperatures (46°C, 48°C and 50°C), and the temperature that produced an average pain intensity score closest to 50 on a 0-100 visual analogue scale (VAS) was used for the test stimulus in the CPM protocol completed on that day. This process was repeated each day that CPM was assessed to determine the temperature necessary to evoke moderate pain during the test stimuli. Subjects verbally rated the intensity of each thermal pulse on a numerical rating scale from 0 = “no pain” to 100 = “the worst pain imaginable” [19]. We selected SHPR as the test stimulus because evidence suggests that CPM effects are largest for C-fiber mediated pain [21,22]. For analyses purposes, this study used the “5th pain rating” which was the fifth pain rating from the fifth pulse of each trial [23-26], which is considered to represent a simple measure of SHPR assessment [27].

##### Conditioning stimulus (cold-pressor pain)

Subjects were instructed to immerse their surgical side hand (for the clinical cohort), and dominant hand (in healthy cohort) up to the wrist into a cold water bath for up to one minute. The water was maintained at a constant temperature of 8°C by a refrigerated water circulator, and was constantly circulated to prevent warming around the hand.

##### CPM procedure

Participants from both cohorts underwent the CPM assessment with the application of the test stimulus (described above) on the non-surgical side for the clinical cohort, and non-dominant side for the healthy cohort. After 30 s from the last heat stimulus, subjects were instructed to immerse their contralateral hand up to the wrist into the cold water bath (conditioning stimulus). Thirty seconds after hand immersion, subjects were asked to rate the pain intensity (0-100) from the immersed hand, and were instructed to maintain their hand in the water bath for as long as they could tolerate for a maximum of one minute. One minute after the immersion of the hand, a new test stimulus was delivered on the non-surgical side for the clinical cohort, and non-dominant side for the healthy cohort. The protocol was created with consecutive stimuli (test stimulus, then conditioning stimulus, hand removed from water, and then test stimulus). This sequence of delivering the test stimuli, followed by the conditioning stimulus, and ending with re-delivering the test stimuli constituted the first CPM trial (CPM1). After a two-minute rest period, the CPM protocol was repeated in exactly the same manner for a second trial (CPM2).

#### Exercise induced muscle pain (EIMP)

Pain was induced in subjects from the healthy cohort with a shoulder fatigue procedure using a Kin-Com (Chattanooga, TN) isokinetic dynamometer. Upper extremity EIMP is considered a clinically relevant pain model since participants experience increased pain intensity, decreased range of motion, an inflammatory response, altered proprioception, and the use of self-care behaviors [28-32]. Muscle soreness occurs within 24 hours following the shoulder fatigue procedure with maximal soreness typically lasting 48 hours from the time of the procedure [28]. Subjects typically experience decreasing levels of muscle soreness three to five days post-shoulder fatigue protocol.

Maximum voluntary isometric contraction (MVIC) was determined by having the participants perform external rotation contractions with maximal effort while receiving verbal encouragement during the contractions. The MVIC was calculated by averaging the peak force from the middle 3 repetitions, a method with documented reliability from previous studies [33,34]. After MVIC was calculated, participants completed eccentric/concentric external rotation repetitions to induce muscle fatigue. Previously established procedures were utilized to induce pain in this cohort, which is described in more detail in our previous studies [15,16].

#### Pain intensity

These data were included to describe the effects of clinical pain (surgical cohort) and exercise-induced shoulder pain (healthy cohort) on CPM stability. Pain intensity in both cohorts was assessed with the Brief Pain Inventory (BPI) questionnaire [35], which includes a numerical rating scale (NRS) for pain intensity. Subjects from the clinical cohort (before and 3 months after surgery) and from the healthy cohort (after EIMP protocol) rated their pain intensity over three conditions, the present pain intensity, the worst pain intensity over the past 24 h, and the best pain intensity over the past 24 h. These 3 ratings were averaged for use in data analyses [36,37].

### Testing sequence

Patients from the clinical cohort had a baseline examination (baseline) to collect demographic data, shoulder pain intensity, and quantitative sensory testing 72 to 24 hours before the surgery. They were reassessed 3 months after the surgery (3 months). Subjects from the healthy cohort were scheduled to come to the testing facility on day 1, day 3, and day 5. On Day 1, subjects had a baseline examination to collect demographic data, shoulder pain intensity, and quantitative sensory testing (CPM and pain threshold) in a pain free state. Subjects completed strength testing, followed by eccentric/concentric external rotation repetitions to induce muscle fatigue. Collection of quantitative sensory testing and shoulder pain intensity was repeated on days 3 and 5 following EIMP induction.

### Data analysis

Data analysis was performed in SPSS, Version 19.0 at alpha level of 0.05. Descriptive statistics (mean, standard deviation) were calculated for all variables. The distributions of variables were tested by visual examination and with Kolmogorov-Smirnov test before use in analysis.

#### Calculations for CPM

For analysis purposes on CPM, we followed recent recommendations [4] on presenting results and calculation of CPM using the absolute difference for CPM and the percent change. The “absolute difference” for CPM, was calculated by the difference between test stimulus before the application of conditioning stimulus (pre CPM), minus the test stimulus after the application of conditioning stimulus (post CPM). The “percent change” for CPM was calculated as follows:

$$\left[\left(\mathrm{post}\;\mathrm{CPM-pre}\;\mathrm{CPM}\right)/\mathrm{pre}\;\mathrm{CPM}\right]*100$$

For each session there were two absolute difference and two percent change scores for CPM variables; one from the first CPM trial (CPM trial 1) and one from the second CPM trial (CPM trial 2). CPM trials were repeated before and 3 month after the surgery for the clinical cohort, and on three different days (Days 1, 3, and 5) for the healthy cohort.

#### Clinical cohort

Stability analyses for CPM measures included intraclass correlations (ICC’s) for the absolute difference of CPM and the percent change of CPM only within session (trial 1 and trial 2). These results were reported with the appropriate coefficient and 95% confidence interval (CI). From these data the standard error of measurement (SEM) was calculated for each measure using a previously described method [standard deviation*√(1 – test rest reliability coefficient)] [38-40]. The minimal detectable change (MDC95) was calculated using a previously described method (1.96*SEM*√2) [41]. The stability estimates (SEM and MDC95) provide an idea of how much individual change is necessary before measurement error is likely to have been exceeded. To determine whether sex influences CPM stability, ICC’s stratified by sex were also reported.

Repeated measures ANOVAs were used to assess the effect of trial (trial 1 and trial 2) on within session for the absolute difference of CPM. Next, shoulder pain intensity was included as a covariate to investigate the impact that pain intensity had on CPM stability. The analysis was performed separately for each session of the clinical cohort (session 1 and 3 months).

#### Healthy cohort

Stability analyses for CPM measures included intraclass correlations (ICC’s) for the absolute difference of CPM and the percent change of CPM within session (trial 1 and trial 2) and between sessions (day 1, day 3, and day 5). As in the clinical cohort, these results were reported with the appropriate 95% CI, SEM, and the MDC_95._ ICC’s stratified by sex were also reported.

Repeated measures ANOVAs were used to assess the effect of trial (trial 1 and trial 2) on within session for the absolute difference of CPM. The analysis was performed separately for each session of the healthy cohort (day 1, day 3, and day 5). In addition, repeated measures ANOVA were used to assess the effect of time (day 1, day 3, and day 5) on the absolute difference of CPM with and without the inclusion of shoulder pain intensity as a covariate to investigate the impact that pain intensity had on CPM stability on between sessions.

## Results

### Subjects

A total of 134 subjects from the clinical cohort, and 190 subjects from the healthy cohort recruited from March 2009 to May 2012 were included in this analysis. Descriptive statistics for the measures are summarized in Table 1 for the clinical and healthy cohorts. Absolute change and percent change of CPM stratified by sex are summarized in Table 2 for both cohorts. All variables were found to approximate a normal distribution by visual examination and Kolmogorov-Smirnov test (*p* > 0.05), and were therefore deemed appropriate for our planned parametric analyses.

**Table 1:** Demographic characteristics, CPM ratings, absolute change and percent change of CPM for clinical and healthy cohorts

**Clinical cohort**	**Mean (SD)**	**Pain rating for test stimulus**	**Pain rating for conditioning stimulus**	**Absolute change of CPM**	**Percent change of CPM**
Age	43.83 (17.80)				
Sex (Female)	47 (35.1%)				
(Male)	87 (64.9%)				
BPI sess 1 (pre surgery)	3.28 (2.31)				
BPI sess 2 (3 months)	1.56 (1.55)				
Pre CPM trial 1 (pre surgery)		28.75 (23.39)	64.30 (27.39)	8.11 (11.78)	23.9%
Post CPM trial 1 (pre surgery)		20.38 (20.99)			
Pre CPM trial 2 (pre surgery)		23.69 (19.99)	62.81 (27.71)	4.66 (8.23)	22.9%
Post CPM trial 2 (pre surgery)		18.96 (19.69)			
Pre CPM trial 1 (3 months)		23.86 (17.35)	62.69 (27.56)	6.51 (9.33)	36.8%
Post CPM trial 1 (3 months)		17.45 (17.81)			
Pre CPM trial 2 (3 months)		19.51 (16.60)	62.13 (27.73)	4.18 (10.02)	9.6%
Post CPM trial 2 (3 months)		15.33 (16.00)			
**Healthy cohort**					
Age	23.02 (6.04)				
Sex (Female)	116 (61.1%)				
(Male)	74 (38.9%)				
BPI day 1	0.45 (0.77)				
BPI day 2	2.05 (1.04)				
BPI day 3	2.44 (1.71)				
BPI day 5	1.23 (1.21)				
Pre CPM trial 1(day 1)		20.81 (20.45)	50.19 (27.77)	9.06 (11.51)	48.4%
Post CPM trial 1 (day 1)		11.75 (14.91)			
Pre CPM trial 2 (day 1)		18.09 (18.87)	50.88 (27.29)	7.08 (10.35)	46.4%
Post CPM trial 2 (day 1)		11.01 (15.14)			
Pre CPM trial 1 (day 3)		20.30 (19.43)	53.97 (27.66)	9.08 (12.74)	46.6%
Post CPM trial 1 (day 3)		11.21 (14.54)			
Pre CPM trial 2 (day 3)		17.66 (18.46)	52.70 (27.11)	6.99 (11.09)	41.0%
Post CPM trial 2 (day 3)		10.67 (13.52)			
Pre CPM trial 1 (day 5)		19.97 (20.61)	54.37 (28.05)	8.60 (13.38)	41.7%
Post CPM trial 1 (day 5)		11.37 (14.24)			
Pre CPM trial 2 (day 5)		16.95 (18.04)	51.96 (27.98)	6.27 (9.99)	39.6%
Post CPM trial 2 (day 5)		10.69 (14.39)			

**Table 2:** Absolute change and percent change of CPM stratified by sex for clinical and healthy cohorts

	**Females**		**Males**	
**Clinical cohort**	**Absolute change**	**Percent change**	**Absolute change**	**Percent change**
CPM trial 1 (pre surgery)	8.56 (11.39)	34.1%	8.86 (12.04)	18.9%
CPM trial 2 (pre surgery)	3.94 (7.80)	21.1%	5.06 (8.47)	23.8%
CPM trial 1 (3 months)	4.97 (9.22)	40.3%	7.04 (9.37)	35.6%
CPM trial 2 (3 months)	2.72 (7.77)	1.2%	4.69 (10.70)	12.3%
**Healthy cohort**				
CPM trial 1 (day 1)	9.11 (11.56)	44.7%	8.96 (11.50)	54.0%
CPM trial 2 (day 1)	7.42 (11.98)	41.5%	6.55 (7.10)	54.0%
CPM trial 1 (day 3)	10.05 (13.87)	50.9%	7.62 (10.67)	39.6%
CPM trial 2 (day 3)	8.11 (13.13)	41.9%	5.27 (6.61)	39.7%
CPM trial 1 (day 5)	9.87 (14.81)	37.8%	6.66 (10.67)	47.8%
CPM trial 1 (day 5)	6.57 (10.17)	38.6%	5.81 (9.77)	41.2%

### Clinical cohort

Table 3 shows stability results from the clinical cohort within session (trial 1 vs trial 2). Estimated ICC’s for the absolute difference and percent change of CPM stratified by sex are reported in Table 4. In the clinical cohort only females had ICC values approaching the commonly used point estimate in the literature of 0.70 [42]. It should be noted that for males the within session stability at baseline did not exceed the recommended ICC’s threshold score of 0.50 (for the absolute difference) [42].

**Table 3:** Stability estimates for CPM within and between sessions for the overall sample

	**Clinical cohort**	**ICC’s for the absolute difference (95% CI)**	**ICC’s for the percent change (95% CI)**	SEM*	MDC*
Within session	CPM trial 1-CPM trial 2 (baseline)	0.54 (0.34-0.68)	0.42 (0.16-0.59)	6.79	18.82
	CPM trial 1-CPM trial 2 (3 months)	0.62 (0.43-0.74)	0.07 (-0.51-0.36)	5.96	16.52
	**Healthy cohort**				
Within session	CPM trial 1-CPM trial 2 (day 1)	0.66 (0.55-0.75)	0.60(0.46-0.70)	6.37	17.66
	CPM trial 1-CPM trial 2 (day 3)	0.72 (0.62-0.79)	0.55 (0.39-0.67)	6.30	17.46
	CPM trial 1-CPM trial 2 (day 5)	0.70 (0.60-0.78)	0.64 (0.51-0.73)	6.40	17.74
Between session	CPM trial 1 (day1)-CPM trial 1 (day3)-CPM trial 1 (day5)	0.71(0.62-0.77)	0.61 (0.49-0.70)	6.75	18.71
	CPM trial 2 (day1)-CPM trial 2 (day3)-CPM trial 2 (day5)	0.68 (0.60-0.76)	0.59 (0.46-0.69)	5.93	16.44

*SEM and MDC were calculated using ICC’s for the absolute difference of CPM.

**Table 4:** Stability estimates for CPM within and between sessions stratified by sex for clinical and healthy cohorts

		**Females**				**Males**			
	**Clinical cohort**	**ICC’s for the absolute difference (95% CI)**	**ICC’s for the percent change (95% CI)**	SEM*	MDC*	**ICC’s for the absolute difference (95% CI)**	**ICC’s for the percent change (95% CI)**	SEM*	MDC*
Within session	CPM trial 1-CPM trial 2 (baseline)	0.63 (0.33-0.80)	0.57 (0.19-0.78)	5.83	16.16	0.49 (0.21-0.67)	0.39 (0.05-0.61)	7.33	20.32
	CPM trial 1-CPM trial 2 (3 months)	0.75 (0.43-0.89)	0.73 (0.36-0.89)	4.25	11.78	0.58 (0.32-0.73)	0.41 (-0.03-0.66)	6.50	18.02
	**Healthy cohort**								
Within session	CPM trial 1-CPM trial 2 (day 1)	0.60 (0.42-0.72)	0.55 (0.34-0.69)	7.45	20.65	0.79 (0.67-0.87)	0.66 (0.45-0.79)	4.26	11.81
	CPM trial 1-CPM trial 2 (day 3)	0.71 (0.57-0.80)	0.61 (0.42-0.74)	7.28	20.18	0.74 (0.58-0.84)	0.52 (0.22-0.71)	4.40	12.20
	CPM trial 1-CPM trial 2 (day 5)	0.64 (0.47-0.75)	0.74 (0.61-0.82)	7.49	20.76	0.83 (0.73-0.89)	0.48 (0.13-0.68)	4.21	11.66
Between session	CPM trial 1 (day1)-CPM trial 1 (day3)-CPM trial 1 (day5)	0.65 (0.51-0.75)	0.62 (0.47-0.73)	7.93	21.98	0.82 (0.73-0.88)	0.60 (0.40-0.75)	4.64	12.86
	CPM trial 2 (day1)-CPM trial 2 (day3)-CPM trial 2 (day5)	0.63 (0.49-0.74)	0.61 (0.46-0.73)	7.15	19.82	0.82 (0.74-0.88)	0.58 (0.35-0.74)	3.32	9.20

*SEM and MDC were calculated using ICC’s for the absolute difference of CPM.

**Figure 1:**
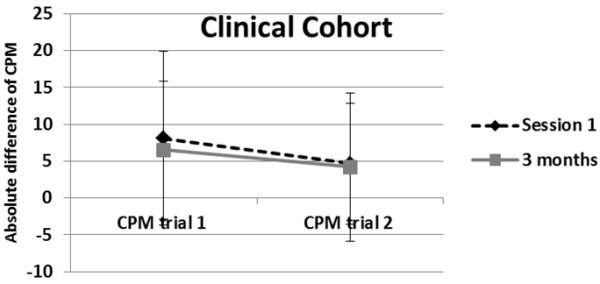
Significant difference between the absolute difference of within session (trial 1 vs trial 2) before the surgery (session 1) and 3 months after the surgery for the clinical cohort.

In addition, analysis revealed that the shoulder pain intensity decreased significantly [F(1, 101) = 65.83; p < 0.001] from the pre surgical time point (mean = 3.26; SD = 2.31) to 3 months post-surgery (mean = 1.52; SD = 1.56). Repeated measures ANOVA showed a significant difference on trial 1 and trial 2 for the absolute difference of CPM within session 1 (before surgery) [F(1,122) = 10.55, p < 0.01]. As expected, the first CPM trial (mean = 9.05, SD = 11.79) produced significantly greater inhibition as compared to the second CPM trial (mean = 4.69, SD = 8.29) (Figure 1). After including pain intensity as a covariate no differences were found from previous results, indicating that the difference between both trials of CPM within the same session was not affected by amount of shoulder pain.

### Healthy cohort

Table 3 shows stability results from the healthy cohort for within session (trial 1 vs trial 2), and between session (day 1, day 3, day 5). Estimated ICC’s for the absolute difference and percent change of CPM stratified by sex are reported in Table 4. Males from the healthy cohort had ICC’s values considered as high scores (for the absolute difference) [42], whereas females had moderate ICC’s [42].

**Figure 2:**
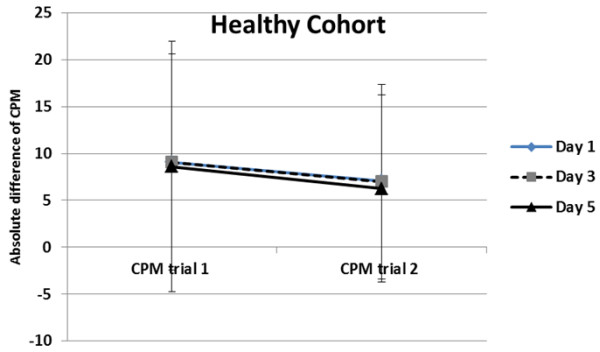
Significant difference between the absolute difference of within session (trial 1 vs trial 2) on day 1, day 3, and day 5 for the healthy cohort.

**Figure 3:**
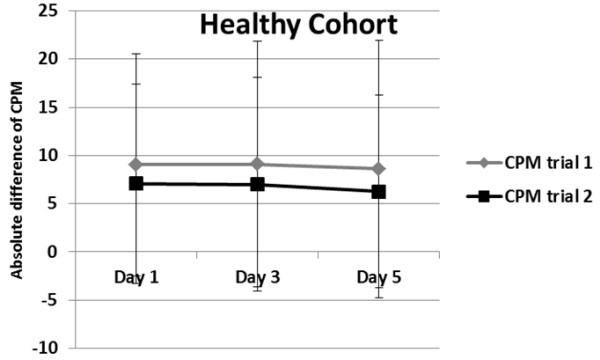
Significant difference between the absolute difference of between sessions (day 1, day 3, and day 5) for the healthy cohort.

Consistent with our previous studies [15,16], induced shoulder pain intensity changed between day 1, day 3, and day 5 [F(2, 374) =178.19; p < 0.001] increasing significantly from day 1 (mean = 0.45; SD = 0.77) to day 3 (mean = 2.45; SD = 1.72), and decreasing significantly from day 3 to day 5 (mean = 1.23; SD = 1.21). Repeated measures ANOVA showed a significant difference between trial 1 and trial 2 for the absolute difference of CPM within day 1 [F(1,189) = 6.16, p = 0.01]. As expected, the first CPM trial (mean = 9.06, SD = 11.51) produced significantly greater inhibition as compared to the second CPM trial (mean = 7.10, SD = 10.34) (Figure 2). These significant differences were maintained within day 3 and day 5 (Figure 3). There was no significant interaction term between CPM trial and pain intensity level (only day 3 and day 5) (p > 0.05), indicating that the difference between both trials of CPM within the same session was not affected by pain intensity.

The between session stability (day 1, day 3, and day 5) of CPM trial 1 [F(2,364) = 0.18, p = 0.84], did not change significantly over time (Figure 3). In exploring whether shoulder pain intensity influenced CPM between session stability, results show that there was no interaction term between time (day1, day 3, day 5) and pain intensity [F(2,362) = 1.89, p = 0.15], indicating that the between session stability of CPM was not affected by subject’s pain intensity. These results did not differ when exploring CPM trial 2.

## Discussion

The present study investigated whether gender and pain intensity influence CPM stability in two different musculoskeletal pain models. Our findings suggest that the stability of CPM did not appear to be related to shoulder pain intensity in surgical or exercise induced pain cohorts, however the stability of CPM within and between days differed by sex. Reliability is an essential property of any measurement that needs to be established before the measurement can be used widely in clinical settings. However, psychometric studies of CPM have not been widely reported [11,43]. The data reported in this paper are novel because to our knowledge, this is the first study to investigate stability of CPM using the same protocol in subjects with 2 different forms of musculoskeletal pain.

We were interested in determining if the inhibition produced by the conditioning stimulus was reliable within the same session (trial 1 and trial 2) in a cohort of patients before and after shoulder surgery (clinical pain model), and in a healthy cohort before and after pain induction (acute pain model). This is an important issue because a measure with poor reliability is unlikely to be valid [44]. Using the most common paradigm to evoke endogenous pain inhibition [4,5,45], CPM proved to suppress SHPR as a test stimulus, such that there was a significant inhibition in trial 1 and in trial 2 (within session) in both cohorts. As expected there was less inhibition in trial 2 compared with trial 1 in both cohorts and across both sessions. The starting points in trial 2 (Pre CPM) were lower than trial 1 (Table 1), producing potentially less room for an inhibitory effect (floor effect). This could be explained by lingering inhibition from the first trial, suggesting an inadequate resting period between trials, and corroborating that the CPM inhibitory effect may last longer than 2 minutes, as other authors have previously suggested [5,7,10]. Therefore, future studies should consider a longer rest period if the goal is to have repeated assessment of CPM within the same session.

Stability of CPM was also assessed between sessions. This enabled us to determine if pre and post-surgery changes on pain intensity (for the clinical cohort), and changes on pain intensity during one week (in the healthy cohort), impacted the stability of the CPM paradigm in a controlled situation. While the shoulder pain intensity changed over time (surgical procedure significantly decreased the amount of pain 3 months after the surgery, and EIMP procedure in the healthy cohort induced a significant amount of pain over 5 days), the amount of inhibition remain relatively constant over time, showing that stability of CPM seems not to be affected much by a short period of time and not to be affected by acute or sub-acute changes on pain intensity. The moderate stability of CPM in two musculoskeletal pain models under different conditions of pain intensity, corroborate the assumption that CPM represents a moderately stable response that is largely independent of changes in pain intensity. Even though the purpose of our study was not directly to compare both cohorts (clinical vs healthy), it is interesting to mention that the conditioning stimulus (cold water bath) produced a significant and comparable pain inhibition in both cohorts. These results could suggest that the ongoing pain in the clinical cohort and the induced pain in the healthy cohort did not affect the mechanisms of CPM, reinforcing the idea of the moderately stable nature of this measure, and confirming using cold water bath as a conditioning stimulus as was previously recommended [4,5]. In terms of CPM calculations, our results further confirm the importance of reporting the absolute and percent change when reporting CMP [4]. Not surprisingly, the ICC’s calculated with the percent change had lower reliability values. This could be explained by the different information that each calculation provides, where the absolute difference represents the absolute magnitude of change, and the percent change accounts for the subject’s baseline level of sensitization.

Regarding the influence of sex on CPM stability within and between sessions, the present study revealed that the stability of CPM within and between days differed by sex (Tables 2 and 4). A number of studies using widely different methodologies have investigated sex differences in experimental pain sensitivity [12] using CPM, or other paradigms. Some researchers suggest less efficient CPM in women than men [14,26,46] while other studies did not detect sex differences in CPM [47,48]. Several factors have been proposed to explain sex differences; however the stability of this experimental pain measure across sexes had not been explored before. In this study, ICC’s stratified by sex showed that males from the clinical cohort showed lower scores than females (within session), and the females from the healthy cohort showed lower scores than males (within and between session for the absolute difference). Furthermore, the standard error of measurement (SEM) is directly related to the reliability of a test; that is, the larger the SEM, the lower the reliability of the test and the less precision in the measures taken and scores obtained. Consequently, our results showed a large difference on SEM between males and females, where males from the healthy cohort and females from the clinical cohort had lower levels of standard error of measurement indicating higher levels of score consistency.

The results of the present study in two musculoskeletal pain models suggest that the most stable situation to use a CPM paradigm is in females from the clinical cohort (where changes need to exceed a MDC of 16 to exceed measurement error) and males from the healthy cohort (where changes need to exceed a MDC of 12 to exceed measurement error). This has a direct clinical implication because whether sex affects reliability of CPM, or whether the amount of measurement variation differs across sex may lead to biased clinical results and biased clinical implications. This differences on CPM stability between sex could potentially explain the contradictory results in the literature using CPM as a measure of central pain processing [3,9,12,49,50], because a lack of reliability does potentially limit the overall validity of a measure. A speculative explanation for our results could be found on differences on psychological factors associated with this experimental pain measure [1,51,52], differences in expectation between sex [53], effect of distraction between sex [26], menstrual cycle effect [54], however future clinical studies need to explore whether these factors directly affect CPM stability.

Previous studies investigating endogenous pain modulation in chronic pain populations have shown a potential deficiency of pain inhibitory system [3,6,7,10,12,26,50,55]. It has also been shown that CPM could be a predictor for post operative pain and potentially sensitive to changes in the central nervous system [3,50]. However, since CPM is a proxy measure of central pain inhibitory process, t, it is particularly important to estimate the error associated with this commonly used measure in order to be useful in research and clinical decision making. After performing a reliability analysis in these two cohorts and establishing that CPM is a measure moderately stable independent of changes in pain intensity, we are in a better stage to use this measure in clinical settings (from a reliability stand point). If we think that CPM is a proxy measure of central pain inhibitory system, with a moderate reliability we may speculate that higher stability may be expected when assessing a population with chronic pain (which may have less variability in central sensitization), however future studies need to be performed to test this hypothesis.

Some important limitations of this study will need to be addressed by future research. First, this investigation was part of a larger study; therefore the procedures were not designed solely for the purpose of establishing CPM stability. Second, this investigation would have been enhanced by adding a control condition where CPM testing was repeated on a second pain-free day. In addition, even though the purpose of the study was not to assess the reliability of CPM in the clinical cohort before and after the surgery, our within session analysis could be affected by the effect of drugs. Future studies should consider the potential effect of drugs before and after the surgery. Lastly, some authors have reported significant sex differences in pain report associated with experimenter sex. The present study examined the effect of sex on CPM, however we did not control for experimenter sex effect, menstrual cycle phases or contraceptive use. Future studies should control for these factors, and should investigate the influence of other relevant demographic characteristic (such as race or ethnicity) on CPM stability.

Despite these limitations, the current study represents a novel contribution to the literature by identifying factors that influence CPM stability in two different musculoskeletal shoulder pain models. Evidence suggests that altered central processing of noxious stimuli might be relevant in the pathogenesis of pain disorders [55-58]. However, establishing the stability of a measurement is essential before consider CPM a clinically useful measure. Our results suggest that 1) CPM stability is not related to changes in pain intensity, and 2) there are sex differences for CPM stability within and between days. Applying these results to clinical psychophysics, our study suggest that the fluctuation of shoulder pain intensity did not significantly affect CPM stability, this could reiterate the moderately stable nature of this construct and the relative consistency of this commonly used experimental pain measure.

## Conclusions

This study assessed whether shoulder pain intensity and gender influence CPM stability in two musculoskeletal pain models. Our results provide evidence that the fluctuation of pain intensity did not significantly influence CPM stability. In addition, there are sex differences for CPM stability within and between days, where the more stable situations for CPM were females from the clinical cohort and males from the healthy cohort.

## Abbreviations

(QST): Quantitative sensory testing; (CPM): Conditioned pain modulation; (EIMP): Exercise induced muscle pain; (MVIC): Maximum voluntary isometric contraction; (SLAP): Superior labrum from anterior to posterior; (VAS): Visual analogue scale; (NRS): Numerical rating scale; (BPI): Brief pain inventory; (ICC): Intraclass correlations.

## Competing interest

The authors declare that they have no competing interest.

## Authors’ contribution

CV, contributed to conception, design, data analyses, data interpretation, and drafting the article. LK, contributed to conception, design, and drafting the article. RF, contributed to data interpretation and critically revising it for intellectual content. SG procured the funding, contributed to conception, design, analyses and interpretation of data. All authors critically revised the manuscript and discussed the results and commented on it. All authors read and approved the final manuscript.

## Pre-publication history

The pre-publication history for this paper can be accessed here:

http://www.biomedcentral.com/1471-2474/14/182/prepub
